# Fourier transform infrared spectroscopy as a non-destructive method for analysing herbarium specimens

**DOI:** 10.1098/rsbl.2022.0546

**Published:** 2023-03-22

**Authors:** M. Barnes, J. Sulé-Suso, J. Millett, P. Roach

**Affiliations:** ^1^ Department of Chemistry, Loughborough University, Loughborough, UK; ^2^ Department of Geography and Environment, Loughborough University, Loughborough, UK; ^3^ School of Pharmacy and Bioengineering, Guy Hilton Research Centre, Keele University, Keele, UK; ^4^ Cancer Centre, University Hospitals of North Midlands, Stoke-on-Trent, UK

**Keywords:** Fourier transform infrared spectroscopy, herbarium, non-destructive, principal component analysis, hierarchical cluster analysis

## Abstract

Dried plant specimens stored in herbaria are an untapped treasure chest of information on environmental conditions, plant evolution and change over many hundreds of years. Owing to their delicate nature and irreplaceability, there is limited access for analysis to these sensitive samples, particularly where chemical data are obtained using destructive techniques. Fourier transform infrared (FTIR) spectroscopy is a chemical analysis technique which can be applied non-destructively to understand chemical bonding information and, therefore, functional groups within the sample. This provides the potential for understanding geographical, spatial and species-specific variation in plant biochemistry. Here, we demonstrate the use of mid-FTIR microspectroscopy for the chemical analysis of *Drosera rotundifolia* herbarium specimens, which were collected 100 years apart from different locations. Principal component and hierarchical clustering analysis enabled differentiation between three main regions on the plant (lamina, tentacle stalk and tentacle head), and between the different specimens. Lipids and protein spectral regions were particularly sensitive differentiators of plant tissues. Differences between the different sets of specimens were smaller. This study demonstrates that relevant information can be extracted from herbarium specimens using FTIR, with little impact on the specimens. FTIR, therefore, has the potential to be a powerful tool to unlock historic information within herbaria.

## 1. Introduction

Herbaria are collections of dried plant specimens, which can date back hundreds of years (from *ca* 1600) and hold huge potential for understanding global environmental and ecological change. Hundreds of millions of specimens are stored worldwide, with approximately 390 million housed in 3100 herbarium collections (https://sweetgum.nybg.org/science/). Rapid advances in cataloguing and digitization of records are revolutionizing the use of herbaria; their potential is only just starting to be realized [1]. Herbarium specimens are biological time capsules providing data that can address environmental and ecological questions over tens to hundreds of years, providing unique insight into environmental and ecological processes from the start of industrialization [2,3]. Methods for the chemical analysis of herbarium specimens can be powerful methodologies for understanding variability within and between species and impacts of environmental changes on plants, and the current limitations of destructive testing methods [4,5]. Measurement of plant morphology, isotope ratios, element concentrations, species identity and DNA have been used to study the impacts of pollution [6,7], climate change, biological invasions and land-use changes. Herbarium specimens are irreplaceable so analysis of specimens must not impact on their value for future research [8]. Measurement of phenology, phenotype and species identity can be completed using non-destructive approaches. Molecular analysis, however, often requires the destruction of sensitive and delicate plant tissue. There is, therefore, a need to develop non-destructive sampling approaches to maximize the extraction of information from historical herbarium samples.

Fourier transform infrared (FTIR) spectroscopy is a rapid, inexpensive, non-destructive method for assessing chemical characteristics of biological samples. This technique measures the interaction of infrared radiation with molecular bonds to provide information on the biochemical compounds in a sample. In plant tissues FTIR has been used to measure the impact on plants of differences in, for example, nutrient availability, water stress, temperature stress, heavy metals and disease [9]. A number of spectral regions give information about lipids, proteins and other more specific bonding within the fingerprint region providing information on, for example, polypeptides, polyphenolics and polysaccarides. Measurement of bonding interactions enables semi-quantitative evaluation of molecular functionality presence (through comparison of spectral regions) and assessment of variation in this within a sample (through comparison of absorption band shape). The ability of FTIR to determine differences in chemical composition between herbarium specimens of the same species provides potential for non-destructive evaluation of spatio-temporal patterns in plant biochemistry. However, to date, FTIR on herbarium specimens has been limited to destructive use and for species identification purposes [10,11]. No one has explored the potential for using FTIR for within-species comparison of herbarium specimens. We, therefore, lack basic understanding of the potential of this approach, and of the feasibility of obtaining relevant, useable data from herbarium specimens.

In this study, our aim was to demonstrate the use of FTIR as a non-destructive technique for analysis of herbarium specimen chemistry. We explored the feasibility of using FTIR for identifying differences between leaf structures and variation between specimens collected at different times in different locations. We used the cosmopolitan carnivorous plant *Drosera rotundifolia* L., which traps prey using adapted leaves presenting a sticky ‘*fly-paper*’ trap. We chose this species owing to its abundance in herbarium collections, wide distribution and because it is a sentinel species being very sensitive to environmental variation [12]. In addition, *D*. *rotundifolia* leaves comprise clearly differentiated structures, which are functionally distinct. The *lamina* is the primary photosynthetic surface, and we know that it changes colour in response to differences in biotic and abiotic conditions [13]. Tentacles arising from the lamina comprise a *tentacle stem* on the end of which is a *tentacle head* which produces a viscous muco-polysaccharide [14]. Since these plants have a distinct life-history strategy, with many chemical elements, this was a good model to evaluate FTIR analysis of any chemical differences within and between individual specimens (young and aged).

## 2. Material and methods

### (a) Sample collection

Four *D. rotundifolia* herbarium specimens were used in this study (table 1, electronic supplementary material, figure S1). Two specimens were plants collected in 2021 from the same location, at the same time by the same collector. The other two specimens were collected from a different location but collected from the same location on the same date in 1934. All samples were in dried form with no observable moisture. Photos of the herbarium sheets are presented in electronic supplementary material, figure S1.

**Table 1.** Herbarium specimen meta data.

specimen ID	date collected	collector	location	habitat	location
A (Young)	27 July 2021	Jonathan Millett	Humberhead Peatlands National Nature Reserve	ombrotrophic bog	53.63203°N, −0.9059°E
B (Young)	27 July 2021	Jonathan Millett	Humberhead Peatlands National Nature Reserve	ombrotrophic bog	53.63203°N, −0.9059°E
C (Old)	7 July 1934	not known	Kärr, Kristinehamns, Sweden	ombrotrophic bog (assumed)	59.12°N, 14.07°E
D (Old)	7 July 1934	not known	Kärr, Kristinehamns, Sweden	ombrotrophic bog (assumed)	59.12°N, 14.07°E

### (b) Fourier transform infrared

**Figure 1.**
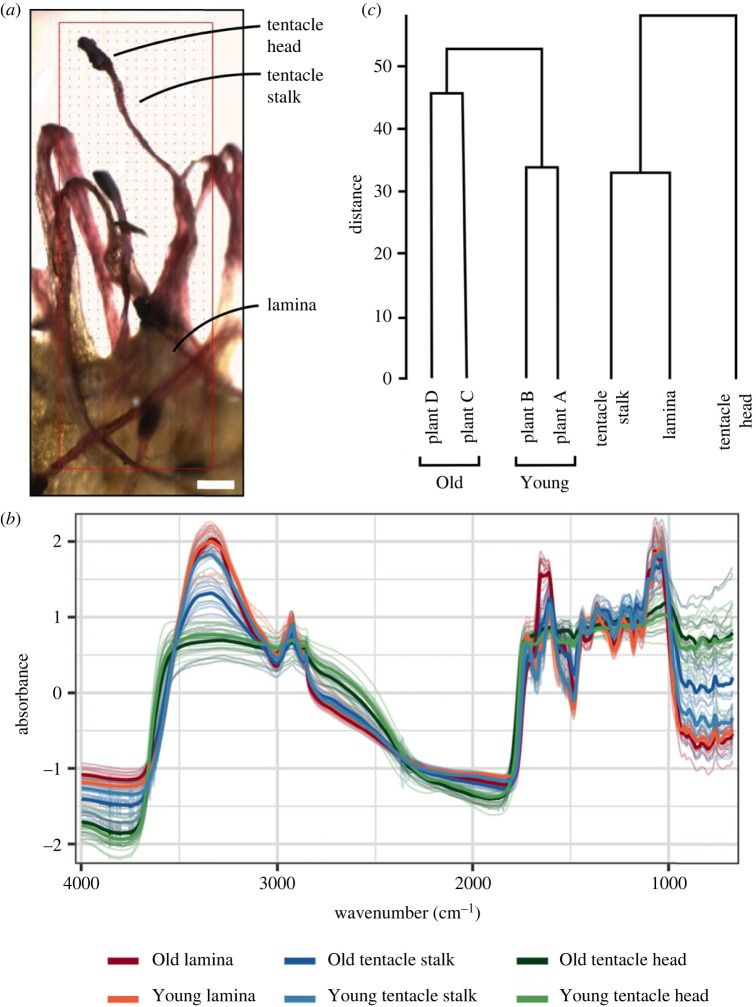
(*a*) Brightfield image showing different regions of the specimens investigated. Scale bar 100 mm. (*b*) Absorbance spectra for different tissue types in different samples groups for ‘Old’ and ‘Young’ specimens. Presented are normalized absorbance across the full spectral range. Solid lines represent the mean spectrum for each group, translucent lines are the spectra for each point within a tissue. (*c*) HCA dendrograms separating between specimens (left) and tissue type (right).

Specimens were carefully removed from the herbarium sheet and placed onto the sample stage. FTIR spectra were taken for each of three tissue types (figure 1: lamina, tentacle stalk, tentacle head), on two leaves, for each of the four specimens. In each area, three replica FTIR spectra maps were produced. Each map consisted of roughly 15 points depending on size of the specimen. Infrared spectra were taken using a Thermo Nicolet iN10mx spectrometer fitted with a liquid nitrogen cooled MCT detector. Spectra were captured in transmission mode, with an 80 × 80 µm aperture size and 4 cm^−1^ resolution. Background spectra were taken in air before each sample, with 64 scans averaged at each map point; samples were suspended enabling direct transmission of IR. Data are available via the Dryad Digital Repository [15].

### (c) Spectra pre-processing

Spectra were obtained directly from Thermo Scientific Omnic Picta (v.9.7.0.39) with map files being split and exported as individual CSVs. Spectral analysis was carried out using Quasar v.1.4.0 (https://quasar.codes). All spectra collected were normalized using SNV (standard normal variate), to account for variations in sample thickness. Spectra were either analysed as a whole from 4000 to 650 cm^−1^, or specifically within the lipid region 3000–2800 cm^−1^; CO_2_ bands were removed from data analysis. Amide regions were not studied separately owing to variation in sample thickness of tentacle heads, particularly in young samples, impacting spectral band shape in that region.

### (d) Multivariate analysis

Principal component (PC) analysis (PCA) was used to highlight key spectral differences by reducing the number of variables in the dataset, being carried out in Quasar v.1.4.0. Ordinations of PC axes and loadings for the different samples were created and assessed visually to establish patterns in differences between FTIR spectra of the samples. To further understand the FTIR spectra, hierarchical cluster analysis (HCA) was carried out for the raw spectra data using Euclidean distance and a max pruning level of 2 in Quasar v. 1.4.0. Linkage was derived from averages.

### (e) Statistical analysis

For statistical analysis the mean of each spectra map was used, to reduce problems with pseudo-replication. Statistical analysis was carried out in Excel v. 2209 (Microsoft Corporation 2022, https://office.microsoft.com/excel) on PC loadings over both the datasets using a two-tail, two-sample *t*-test assuming unequal variances. *p*-Values were adjusted using a Bonferroni correction to account for the number of *t*-tests done. Evidence of spectral differences between samples was considered in terms of the extent of the difference in PC loading scores and the *p*-value for the differences between the two groups.

## 3. Results

### (a) Whole spectra analysis (4000–650 cm^−1^)

All spectra demonstrated broadly similar peaks but these varied in intensity and shape (figure 1*b*). Peak intensity differed between the 3500–2700 cm^−1^ and the 1700–1100 cm^−1^ regions, probably owing to variation in sample thickness. This resulted in truncation of amide band peaks, particularly for young samples. The HCA dendrogram shows differentiation between sample and tissue type on the basis of the full, raw spectra data. HCA segregated the four plants into four separate groups (figure 1*c*). Plants A and B were linked as similar, as were plants C and D. In addition, HCA differentiated tissue types. Lamina and tentacle stalk were more similar to each other than they were to tentacle head.

**Figure 2.**
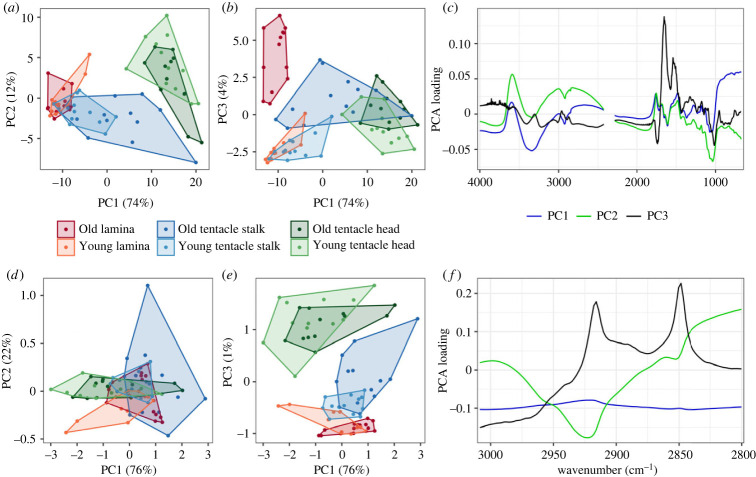
PCA scores and loadings plots for (*a–c*) the whole spectra analysis (4000–650 cm^−1^) and (*d–f*) the lipid specific region (3000–2800 cm^−1^). PCA coordinates on PC axes 1, 2 and 3 are presented for each measured point for different tissue types and different sample categories (details in table 1). PCA loadings plots present the contribution of each wavelength to each PC.

PCA analysis of the whole spectra indicates that the first three PCs accounted for 90% of variance within the spectra. PC1 accounted for 74% of variance, PC2 12% and PC3 4%. The clearest differences in PC scores are between tissue types (figure 2*a*,*b*, table 2). Indicating that the largest amount of variability in FTIR spectra is variation between different tissues. All three tissue types were different from each other, on all three PC axes (table 2). Tissue types are most differentiated along PC1. Differences between ‘Old’ and ‘Young’ specimens are smaller and are differentiated mainly along PC3. PC1 loadings indicate discriminatory components in the amide (N–H ∼ 3500 cm^−1^/1500 cm^−1^ and C=O 1700–1600 cm^−1^), lipid (C–H ∼ 3000–2800 cm^−1^) and further in the fingerprint region (figure 2*c*). This demonstrates that the variability between tissue types is owing to differences between spectra in these regions. PC2 and PC3 show discrimination again in these regions, with PC3 highlighting protein bands as a dominant feature. This demonstrates that variability between different aged specimens is owing to differences in the spectra in these regions.

**Table 2.** Principal component analysis information for sample sub-sets across spectral components. See electronic supplementary material, figure S2 for a graph of *p*-values versus effect size for these results.

		all data			lipids		
		PC1	PC2	PC3	PC1	PC2	PC3
*comparing tissue types*							
lamina versus tentacle stalk	difference	8.11	1.58	1.55	0.35	0.63	0.17
	*t*-value	−15.96	7.53	7.23	−9.94	−2.97	−9.04
	d.f.	541	716	657	791	586	721
	*p*-value	<0.0001	<0.0001	<0.0001	0.002	<0.0001	<0.0001
tentacle stalk versus tentacle head	difference	16.50	4.92	0.40	1.23	1.38	0.05
	*t*-value	−27.63	−12.47	−2.93	8.51	−33.84	3.38
	d.f.	741	511	736	540	730	506
	*p*-value	<0.0001	<0.0001	0.06	<0.0001	<0.0001	0.01
lamina versus tentacle head	difference	24.61	3.34	1.14	0.88	2.00	0.011
	*t*-value	60.03	8.96	−5.68	−6.322	60.91	9.77
	d.f.	520	420	553	481	464	581
	*p*-value	<0.0001	<0.0001	<0.0001	<0.0001	<0.0001	<0.0001
*comparing specimens of different ages*							
Old versus Young lamina	difference	0.12	0.30	6.42	0.73	0.13	0.11
	*t*-value	−0.31	−1.28	37.05	6.70	−5.30	5.53
	d.f.	361	379	332	374	287	366
	*p*-value	1.00	1.00	<0.0001	<0.0001	<0.0001	<0.0001
Old versus Young tentacle head	difference	0.93	2.20	1.47	−0.48	0.16	0.03
	*t*-value	1.26	−3.12	10.04	1.87	−2.58	2.81
	d.f.	303	327	341	321	302	324
	*p*-value	1.00	0.07	<0.0001	1.00	0.37	0.19
Old versus Young tentacle stalk	difference	8.41	0.83	3.24	0.61	0.54	0.04
	*t*-value	9.79	−2.37	21.19	4.50	11.14	1.31
	d.f.	325	357	385	394	290	295
	*p*-value	<0.0001	0.65	<0.0001	0.0003	<0.0001	1.00

### (b) Lipid region analysis (3000–2800 cm^−1^)

PCA analysis of the lipid region alone indicated differences between samples and tissues (table 2). The largest differences are between the different tissue types (figure 2*d*,*e*). All three tissue types were different from each other, on all three PC axes. ‘Old’ and ‘Young’ specimens are differentiated for the lamina area on all three PC axes, for the tentacle stalk on PCs 1 and 2, and not at all for the tentacle head area. For PC1 differentiation is across the entire lipid region spectra, for PC3 differentiation is owing to peaks at 2850 and 2910 cm^−1^. This indicates that variability between the specimens is accounted for by the range of lipids detected by FTIR analysis.

## 4. Discussion

Our aim was to demonstrate FTIR as a non-destructive method for the analysis of herbarium specimen chemistry. We found clear spectral differences between the three plant tissue types (lamina, tentacle stem and tentacle head) and between specimens collected nearly a century apart from different locations. We found evidence of differences between tissue types and specimens primarily in the lipid region associated with fats, fatty acids, waxes and lipids (3000–2800 cm^−1^), and in the amide region associated with proteins (approx. 1700–1500 cm^−1^). Tentacle heads were quite thick in some specimens resulting in semi-truncated spectra in the amide region. Little variation was observed directly in relation to specimen age, except for the slightly less prominent band at approximately 1710 cm^−1^ associated with a carbonyl likely from cellulose. This type of observation has been reported before in tree bark studies, where retting may have resulted in reduction of cellular material [16]. With main cellulose banding in the fingerprint region remaining, this suggests that some breakdown of specimens may have started to occur, reducing the connectivity of tissues rather than complete degradation. This is likely to be a signal relating to specimen age, rather than differences in the living plants.

The non-destructive nature of FTIR is very attractive when dealing with sensitive and limited sample numbers, but FTIR is rarely used for herbarium specimens. Julier *et al.* [10] successfully discriminated between Poaceae (grass) pollen samples at subfamily level, while Lomax *et al.* [11] used FTIR to assess historical levels of UV-B absorbing pigment in plant spore walls from herbarium samples to understand changes in stratospheric ozone concentrations, although this analysis was destructive. Our results demonstrate the potential for this technique to provide non-destructive understanding of differences between herbarium specimens at the biochemical scale. The data obtained from FTIR studies are limited to measuring the chemical functionalities present with a sample, but still provides useful insight into chemical differences between samples, and when applied in combination with other non-destructive techniques such as X-ray fluorescence (XRF) could be quite powerful.

Our study shows that FTIR data can be used to characterize biochemical components within herbarium specimens, indicating chemical differences in different plant tissues, with limited levels of molecular degradation over time. The three tissues we measured support different functions, and so we expected their different biochemistry to result in clear separation in FTIR spectra. We found this to be the case. These differences likely result from C–O and overlapping O–H deformation associated with lignins (approx. 1350–1310 cm^−1^) [17] the sugar ring vibration (approx. 740 cm^−1^) [18], and fats/waxes/lipids (3000–2800 cm^−1^). A wide range of metabolites are involved in plant carnivory [19] and prey capture results in a whole metabolome response, with large changes in hundreds of metabolites [20]. It seems likely, therefore, that the differences between these tissues are representative of some components of adaptation for carnivory. Jasmonates, for example, are particularly important for carnivorous plants because they are likely to signal prey capture and instigate leaf bending to enhance prey retention [19]. Pavlovič *et al.* found that concentrations of Jasmonates increased after prey capture in both *Drosera capensi*s [21] and *Dionaea muscipula* [22]. Such biochemical signatures of carnivory seem likely to be differentiated within leaf structures, and so likely contribute to the differences we found.

Differences between the specimens which were collected from separate locations nearly 100 years apart, were less clear. Age and location confounded, so it is not possible to determine which contributes to these differences, but comparison is useful to highlight the potential for understanding temporal and/or spatial variation in plant biochemistry. The different ages contrasted primarily in amine and carbonyl spectral features, likely resulting from variation in protein or alkanoic amine chemical groups. This could suggest partial degradation of protein molecules in specimens, although spectra show band broadening rather than reduction in intensity with age, possibly indicating denaturing rather than breakdown of the protein chemistry. This change is also positively linked with changes in the lipid region supporting chemical structural change. Mendes Resende *et al.* [23] also showed, using mass spectrometry, that although chemical degradation within specimens is expected, lipids are still present in sufficient quantities to provide meaningful data for understanding differences in specimens which relate to the plants when alive. Age was not able to be clearly discriminated using only the lipid region, supporting the work of others that lipids may not degrade over time sufficiently to be detected through FTIR analysis. This suggests that the lipid region is a suitable focus for comparison of samples of different ages, to understand changes in plant biochemistry over time.

As all samples were dried we interpret the differences found as being associated with the sample itself (i.e. it is age or location) rather than changes owing to storage. Differences in sample preparation and analysis have been shown to result in spectral variation [24] which would not be present in our analysis. Using transmission mapping enabled good interrogation of all samples non-destructively, also being semi-automated. Although all spectra present similar bands, the tentacle heads did not transmit as effectively giving rise to flattened spectra compared to others (as highlighted in figure 1*b*), which is more evident in young specimens, and most prominent for the cellulose peak at approximately 1040 cm^−1^.

The delicate and finite nature of herbarium specimens necessitates the use of non-destructive analysis methods, with these samples providing historical records of environmental impact/change and evolutionary change. Our measurements demonstrate the feasibility of using FTIR to extract meaningful data from *D. rotundifolia* herbarium specimens and show that the data collected differentiates within and between plant tissues, with limited change related to specimen age. Spectra obtained from age-matched similar plant regions were consistent across the sample repeats tested, indicating the robustness of the methodology and potential for further analysis of herbarium specimens. More work is required to understand the potential that FTIR analysis offers for the chemical interrogation of historical plant specimens. Further comparison of large numbers of samples, along with verification using more-sensitive approaches which may require destructive sampling is needed to ensure robust interpretation of FTIR data. Such confirmation of the potential and limitations of FTIR will enable the exploration of the environmental and climate change chemistry locked within these samples.

## Data accessibility

Data freely available Open Acess from the Dryad Digital Repository: https://doi.org/10.5061/dryad.jsxksn0dw [15].

Data being shared as raw spectral files as both original instruments file (Thermo Scientific Omnic Picta .map files) and as .CSV exports for wide dissemination of the source data.

The data are also provided as part of the electronic supplementary material [25].
